# Enhanced Surveillance for White-Nose Syndrome in Bats

**DOI:** 10.3201/eid1803.111751

**Published:** 2012-03

**Authors:** Anne Griggs, M. Kevin Keel, Kevin Castle, David Wong

**Affiliations:** National Park Service, Mammoth Cave, Kentucky, USA (A. Griggs);; Southeastern Cooperative Wildlife Disease Study, Athens, Georgia, USA (M.K. Keel);; National Park Service, Ft. Collins, Colorado, USA (K. Castle);; National Park Service, Albuquerque, New Mexico, USA (D. Wong)

**Keywords:** white-nose syndrome, One Health, Kentucky, Tennessee, Mammoth Cave, National Park Service, disease surveillance, bats, wildlife, fungi

**To the Editor:** White-nose syndrome (WNS) is an emerging fungal disease in bats that was first described near Albany, New York, USA, in February 2006 (*1*). The causative agent, *Geomyces destructans*, is a psychrophilic (cold-loving) fungus that infects the skin of bats and leads to depletion of their fat stores during hibernation (*2*). WNS has caused dramatic cumulative mortality rates (up to 99%) in some winter hibernacula and has killed millions of bats among 6 cave-roosting species in 19 central and eastern US states and 4 Canadian provinces (*3*). In addition, the fungus has been identified in 2 additional US states, although bat deaths have not been associated with it. No evidence has been found that WNS is transmitted from bats to humans, although humans may play a role in translocation of the fungus between caves (*4*,*5*).

Current surveillance for WNS is time- and labor-intensive. Wildlife personnel typically enter caves, inspect hibernacula, and collect bats with clinically compatible signs for testing (*4*). In July 2010, the National Park Service (NPS) Office of Public Health proposed an expanded WNS surveillance strategy that involved using opportunistic sampling of bats already submitted to state public health laboratories for rabies testing; the bats submitted include species known to be susceptible to WNS. The pilot study focused on the region around Mammoth Cave National Park, the world’s longest known cave system and home to 13 bat species (2 endangered), in south-central Kentucky (*6*). At the time of initial discussions, Kentucky was WNS-free, but the bordering state of Tennessee had recently reported its first WNS cases in spring 2010 in a cave system located <130 km from Mammoth Cave. WNS was first detected in Kentucky in April 2011 in Trigg County (180 km from Mammoth Cave) (*7*).

The goals of this pilot study were to 1) enhance WNS surveillance in counties in and near Mammoth Cave and 2) demonstrate a feasible, cost-effective surveillance system. NPS Office of Public Health staff coordinated meetings in Kentucky and Tennessee with representatives from the state departments of wildlife and health and other partnering organizations. Key representatives at one or both of these meetings included the state epidemiologist, the state public health veterinarian, the public health laboratory director, state wildlife biologists, and NPS and state wildlife veterinarians. Also attending both meetings was a veterinary pathologist from the Southeastern Cooperative Wildlife Disease Study (SCWDS) in Athens, Georgia, USA, one of 3 laboratories that test most samples for WNS in the United States. The surveillance concept was well received in both states, and state-specific protocols were developed for submitting rabies-negative bats to SCWDS only during hibernation months (November–April) when WNS is more likely to be detected (*8*). In Kentucky, a memorandum of understanding was drafted that outlined roles and responsibilities of collaborating agencies. The memorandum was reviewed by legal advisors and signed by public health and wildlife officials.

Both protocols outlined key elements of the submission process, including how laboratory personnel were to submit rabies-negative bats to SCWDS for WNS testing (fungal culture, histopathologic examination, and PCR), how bats were to be stored or destroyed after testing, and the chain of communication for reporting test results. Whenever possible, bats were frozen at −20°C within 48 hours following rabies testing, and their muzzles and forearms were left intact to maximize the yield for *G. destructans* and to facilitate species identification. Protocols included additional criteria to improve testing efficiency (e.g., prioritizing submissions on the basis of known WNS-susceptible species or counties where cave-roosting colonies are located). A project-specific version of the standard SCWDS submission form was completed for all samples. All funding and resources were provided in kind by respective agencies.

In October 2010, the Tennessee State Public Health Laboratory submitted 34 rabies-negative bats (archived during January–April 2010, before pilot study discussions) from 18 counties to SCWDS; all were WNS-negative. Twenty-one additional rabies-negative bats from 9 Tennessee counties collected during November 2010–April 2011 also tested negative for WNS. In Kentucky, 64 rabies-negative bats (from 22 counties) were submitted during November 2011–January 2012; all were WNS-negative except 1 bat tested on January 13, 2012, which was the first known WNS-positive bat from Fayette County, a primarily urban area in northern central Kentucky where little cave-based WNS surveillance is conducted. Overall, although the sample of bats tested to date is modest and likely insufficient as a stand-alone surveillance system, these results supplement other data and can inform the development of interventions, prevention messages, and transmission models.

This pilot study highlights several observations and implications. First, it demonstrates that opportunistic testing of rabies-negative bats for WNS can be facilitated between state departments of wildlife and health through interagency collaboration. Second, the surveillance system is low cost and could potentially be expanded to other states where WNS is likely to emerge and where statewide cave-based surveillance is cost-prohibitive. Last, this project showcases a unique interdisciplinary collaboration in wildlife and human health, disease ecology, and environmental stewardship. Such partnerships are championed by the One Health approach (*9*) and are central to the mission of NPS to protect the health of all species and our environment (*10*).
